# Identification of Dutch children diagnosed with atopic diseases using prescription data: a validation study

**DOI:** 10.1007/s00228-015-1940-x

**Published:** 2015-10-09

**Authors:** B Mulder, F Groenhof, L I Kocabas, HJ Bos, TW De Vries, E Hak, CCM Schuiling-Veninga

**Affiliations:** Department of PharmacoEpidemiology and PharmacoEconomics University Centre of Pharmacy, University of Groningen, P.O. Box XB45, A. Deusinglaan 1, 9713 AV Groningen, The Netherlands; Department of General Practice, University of Groningen, University Medical Centre Groningen, Groningen, The Netherlands; Department of Pediatrics, Medical Centre Leeuwarden, H Dunantweg 2, 8934 AD Leeuwarden, The Netherlands; Department of Epidemiology, University Medical Centre Groningen, Hanzeplein 1, P.O. Box 30.001, 9700 RB Groningen, The Netherlands

**Keywords:** Prescriptions, Specificity, Sensitivity, Atopic diseases, Asthma, Eczema, Hay fever

## Abstract

**Purpose:**

The aim of this study is to validate medication proxies for the identification of children diagnosed with atopic disorders that can be applied in various types of epidemiological research.

**Methods:**

Records of 7439 children, aged between 0 and 10 years, in the period 2001 until 2010, were retrieved from the Registration Network Groningen database, a general practitioners database in the north-eastern part of the Netherlands. The sensitivity and positive predictive value (PPV) of 22 medication proxies for the identification of children diagnosed with atopic disorders (asthma, atopic dermatitis, and allergic rhinitis) were computed using the registered diagnoses as gold standards. In addition, different capture periods (1 year, half year, and length of study period) for the detection of prescriptions were tested for all the medication proxies.

**Results:**

The highest PPV (0.84, 95 % CI 0.81–0.87) in combination with a sufficient sensitivity value (0.54, 95 % CI 0.50–0.57) for the identification of children diagnosed with asthma was yielded for the medication proxy, ≥2 prescriptions for anti-asthma medication within 1 year, including 1 inhaled steroid. PPV and sensitivity were even higher in the age group 6–10 years. The proxies designed for the identification of children diagnosed with atopic dermatitis and allergic rhinitis yielded only high PPVs (≥0.75) in combination with low sensitivity values (≤0.22). Altering the capture period for the detection of prescriptions to half a year or the length of the study period only affected sensitivity values.

**Conclusion:**

Children diagnosed with asthma can be identified reliably with a range of medication proxies. The use of prescription data for the identification of children diagnosed with atopic dermatitis and allergic rhinitis is questionable.

## Introduction

Pharmacy databases with large numbers of prescriptions can provide valuable information for observational studies [1]. However, the validity of using prescription data for the identification of children with atopic diseases in epidemiological research has been questioned, especially in children [2–8].

Various efforts have already been made to validate methods for the identification of asthma patients with prescription data [3–8]. A former study of our group investigated the accuracy of the use of several medication proxies for the identification of asthma patients (age 19–49) and showed that asthma patients could be identified reliably from prescription data [5]. However, results from an adult patient population cannot directly be generalized to a population of children, especially not in the case of asthma [9]. Making an asthma diagnosis in children is difficult since objective lung tests cannot be performed in patients under the age of 6 [9]. In addition, children may use anti-asthma medication for other indications than adults (acute bronchitis versus chronic obstructive pulmonary disease) [2]. Recent studies developed a valid method for the identification of asthmatic children (age 4.5–17 years) using prescription data [3, 7, 8]. However, for different study purposes, different accuracy measures (sensitivity, specificity, positive predictive value, negative predictive value) are important and hence various proxies are needed to be developed for the use in various epidemiological studies [10]. In addition, children with asthma often have concomitant allergic diseases like atopic dermatitis or allergic rhinitis, due to common pathogenesis [11]. To our knowledge, only one study has focused on the validation of an identification tool for children with atopic dermatitis and concluded that the identification of children with this disease based on a medication proxy was insufficient [8]. Since only one medication proxy was tested and only positive predictive values were reported, there is still need for the validation of other proxies for atopic dermatitis. In addition, no studies have focused on the identification of children diagnosed with allergic rhinitis.

Therefore, the main objective of this study was to determine reliable medication proxies for the identification of children diagnosed with atopic diseases (asthma, atopic dermatitis, and rhinitis) applicable in different types of epidemiological research.

## Methods

### Setting

This register-based cross-sectional study was performed with data from the Registration Network Groningen (RNG) database. This network was established in 1989 and consists of patient registrations of three general practices with 17 general practitioners (GPs) in the north-eastern part of the Netherlands. The RNG includes a dynamic population with an average annual population of approximately 30,000 patients. Patients were anonymized and identified with a unique patient number. This network contains information about patient characteristics, diagnoses, and prescription records for each patient. Each prescription contains an anatomical therapeutic chemical code (ATC-code), and each diagnosis is described with the International Classification for Primary Care (ICPC) code [12]. GPs are trained specifically to work with the coding system, and coding of ICPC and ATC codes was proven to be accurate in previous studies [7, 13]. In the Netherlands, patients are registered to a single GP, so records in the RNG database can be assumed to be complete for the individual patient [14]. Diagnoses and prescriptions from specialists are included in the database if these are communicated to one of the GPs by a so-called retour letter. More information about the database is described elsewhere [15, 16].

### Study population

Patient records from 01 January 2001 until 31 December 2010 were selected from the RNG database. Patients were included in the study if they had at least one physician encounter (visit, telephone consultation, or prescription request) during the study period and were aged between 0 and 10 years at the moment of encounter.

### Prescription data

Data were obtained for all prescriptions regarding drugs for obstructive airway diseases (ATC R03), dermatological preparations with corticosteroids (ATC D07), other agents for the treatment of dermatitis (ATC D11AH), nasal preparations (R01), and systemic antihistamines (ATC R06) prescribed from 01 January 2001 until 31 December 2010. Twenty-two medication proxies (listed in Table 1) with a capture period of 1 year were designed for the identification of children diagnosed with atopic diseases, according to the Dutch guidelines for general practitioners [17–19]. The accuracy measures sensitivity and PPV were calculated for all medication proxies. Specificity and NPV were not calculated since these values will be artificially high, due to the large number of non-allergic patients included in the study population [5].

**Table 1:** Medication proxies used for the identification of children diagnosed with the atopic disorders asthma, atopic dermatitis, and allergic rhinitis

Indication (ICPC code)	nr	Medication proxies
Asthma (ICPC R96)	1	≥1 prescription for an inhaled anti-asthma drug (ATC R03)
	2	≥1 prescription for an inhaled corticosteroid (ATC R03AK, R03BA)
	3	≥2 prescriptions for an inhaled anti-asthma drug (ATC R03)
	4	≥1 inhaled steroid (ATC R03AK, R03BA) + ≥1 inhaled other anti-asthma drug (ATC R03)
	5	≥3 prescriptions for an inhaled anti-asthma drug (ATC R03)
	6	≥2 prescriptions for an inhaled corticosteroid (ATC R03AK, R03BA)
	7	≥4 prescriptions for an inhaled anti-asthma drug (ATC R03)
	8	≥5 prescriptions for an inhaled anti-asthma drug (ATC R03)
Atopic dermatitis (ICPC S87)	9	≥1 prescription for a dermal steroid (ATC D07)
	10	≥1 prescription for an ointment with an immunosuppressant (ATC D11)
	11	≥2 prescriptions for a dermal steroid (ATC D07)
	12	≥3 prescriptions for a dermal steroid (ATC D07)
	13	≥4 prescriptions for a dermal steroid (ATC D07)
	14	≥5 prescriptions for a dermal steroid (ATC D07)
Allergic rhinitis (ICPC R97)	15	≥1 prescription for a systemic antihistamine (ATC R06)
	16	≥1 prescription for a nasal antihistamine (ATC R01AC)
	17	≥1 prescription for a nasal corticosteroid (ATC R01AD)
	18	≥2 prescriptions for systemic antihistamines (ATC R06), nasal antihistamines (ATC R01AC) or nasal steroids (ATC R01AD)
	19	≥3 prescriptions for systemic antihistamines (ATC R06), nasal antihistamines (ATC R01AC) or nasal steroids (ATC R01AD)
	20	≥2 prescriptions for a nasal corticosteroid (ATC R01AD)
	21	≥4 prescriptions for systemic antihistamines (ATC R06), nasal antihistamines (ATC R01AC) or nasal steroids (ATC R01AD)
	22	≥5 prescriptions for systemic antihistamines (ATC R06), nasal antihistamines (ATC R01AC) or nasal steroids (ATC R01AD)

### Analysis

A recorded diagnosis of either atopic dermatitis (ICPC S87), asthma (ICPC R96), or allergic rhinitis (ICPC R97) during the study period was defined as the gold standard. For each medication proxy, the sensitivity, positive predictive value (PPV), and 95 % confidence intervals (95 % CI) were calculated. In additional analyses, different capture periods for the detection of prescriptions (half year and total study period) were tested for all the medication proxies. Since the PPV is dependent on the prevalence of the indication in a population, a sensitivity analysis was performed for the proxy ≥2 inhaled steroids within a year. In this extra analysis, positive predictive values were calculated for a reasonable range of prevalence numbers of asthma, derived from an ISAAC study into the worldwide trends in asthma prevalence [20]. All analyses were conducted using the IBM SPSS Statistics 20 version.

## Results

In total, 7439 children until the age of 10 years were included in the study. Of these children, 1835 (24.7 %) had a registered diagnosis of an allergic disorder (asthma, atopic dermatitis, or allergic rhinitis) in their records. The diagnosis asthma (ICPC R96) was registered in 809 (10.9 %) children (Table 2), atopic dermatitis (ICPC S87) in 985 (13.2 %) children (Table 3), and allergic rhinitis (ICPC R97) in 419 (5.6 %) children (Table 4). Drugs for obstructive airway disease (ATC R03) were used by 744 (10.0 %) children, dermal corticosteroids (ATC D07) by 1990 (26.8 %) children, and nasal antihistamines (ATC R01AC) or nasal corticosteroids (R01AD) by 518 (7.0 %) children.

**Table 2:** Number of patients aged 0–10 years that received anti-asthma medication, stratified by diagnosis

			Diagnosis						
		Asthma	Other respiratory diagnoses without concomitant asthma						
Type of medication		Asthma ICPC R96 (*N* = 809)	Acute bronchitis CPC R78 (*N* = 1209)	Cough ICPC R05 (*N* = 1439)	Dyspnoe ICPC R02 (*N* = 206)	Wheezing ICPC R03 (*N* = 110)	Chronic bronchitis ICPC R91 (*N* = 13)	Allergic rhinitis ICPC R97 (*N* = 295	Other respiratory symptoms ICPC R04 (*N* = 74)
Medication for obstructive airway diseases (R03) (%)	1381	744 (53.9)	389 (28.2)	335 (24.3)	110 (7.9)	85 (6.2)	6 (0.4)	47 (3.4)	20 (1.4)
Inhaled bronchodilator (R03AC)	1230	671 (54.6)	344 (28.0)	285 (23.2)	105 (8.5)	83 (6.7)	4 (0.3)	45 (3.7)	17 (1.4)
Inhaled corticosteroids (R03AK, R03BA)	659	504 (76.5)	111 (16.8)	94 (14.3)	26 (3.9)	16 (2.4)	1 (1.5)	10 (1.5)	4 (0.6)
Systemic antihistamines (R06)	1332	292 (21.9)	316 (23.7)	462 (34.7)	47 (3.5)	29 (2.2)	1 (0.1)	204 (15.3)	23 (1.7)
Systemic corticosteroids (H02)	172	115 (66.9)	26 (15.1)	18 (10.5)	11 (6.4)	2 (1.2)	0	6 (3.5)	2 (1.2)

Numbers and percentages do not sum up to 100 %, due to multiple medications and diagnoses

*ICPC* International Classification for Primary Care

**Table 3:** Number of patients aged 0–10 years that received medication for the treatment of dermatitis, stratified by diagnosis

				Diagnosis					
		AD	Other dermatologic diseases without concomitant atopic dermatitis						
Type of medication		Atopic dermatitis ICPC S87 (*N* = 985)	Contact dermatitis/other dermatitis ICPC S88 (*N* = 1001)		Local irritated skin/erythemaI CPC S06 (*N* = 379)	Pruritus/Itch ICPC S02 (*N* = 184)	General irritated skin/erythema ICPC S07 (*N* = 194)	Seborric eczema ICPC S86 (*N* = 107)	Diaper rash ICPC S89 (*N* = 214)
Dermal antipruritics (D04)	86	23 (26.7)	16 (18.6)		6 (7.0)	8 (9.3)	3 (3.5)	1 (1.2)	6 (7.0)
Dermal corticosteroids (D07)	1990	789 (39.6)	749 (37.6)		124 (6.2)	78 (3.9)	51 (2.6)	68 (3.4)	65 (3.3)
Dermal immunosuppressants (D11AH)	17	12 (70.6)	2 (11.8)		1 (5.9)	3 (17.6)	0 (0.0)	0 (0.0)	1 (5.9)
Systemic antihistamines (R06)	1332	300 (22.5)	231 (17.3)		98 (7.4)	75 (5.6)	73 (22.0)	22 (1.7)	45 (3.4)

Numbers and percentages do not sum up to 100 %, due to multiple medications and diagnoses

*ICPC* International Classification for Primary Care

**Table 4:** Number of patients aged 0–10 years that received medication for the treatment of allergic rhinitis, stratified by diagnosis

		Diagnosis		
		Allergic rhinitis	Other diseases without concomitant allergic rhinitis	
Type of medication		Allergic rhinitis ICPC R97 (*N* = 419)	Asthma ICPC R97 (*N* = 690)	Sneezing/cold ICPC R07 (*N* = 263)
Nasal preparations (R01)	752	238 (31.6)	99 (13.2)	85 (11.3)
Nasal anti-allergic preparations (R01AC)	162	103 (63.6)	44 (27.2)	6 (3.7)
Nasal steroids (R01AD)	356	152 (42.7)	43 (12.1)	51 (14.3)
Systemic antihistamines (R06)	1332	301 (22.6)	195 (14.6)	58 (4.4)

Numbers and percentages do not sum up to 100 %, due to multiple medications and diagnoses

*ICPC* International Classification for Primary Care

### Validation of the medication proxies for the identification of children diagnosed with asthma

**Fig. 1:**
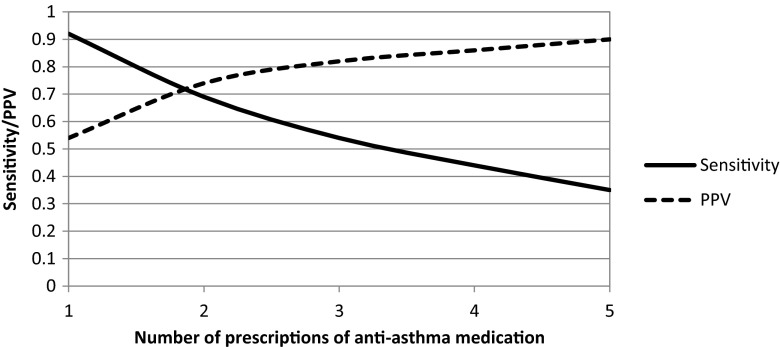
Development of sensitivity and positive predictive value (PPV) with an increasing number of anti-asthma prescriptions within 1 year

In Table 5, the sensitivity and PPV of the eight medication proxies for the identification of children diagnosed with asthma are shown. When a capture period of 1 year was applied, medication proxy 1, receiving ≥1 prescription for any anti-asthmatic drug, yielded the highest sensitivity of 0.92 (95 % CI 0.90–0.94). However, only half of the identified children with this proxy had a registered diagnosis of asthma (PPV 0.54, 95 % CI 0.51–0.57). Table 2 shows that of the children that got prescribed anti-asthma drugs, 28.2 % had a diagnosis of acute bronchitis and/or 24.3 % a diagnosis of cough without a concomitant asthma diagnosis. Of the children who got prescribed inhaled corticosteroids, only 16.8 and 14.3 % had a diagnosis of acute bronchitis or cough without a concomitant asthma diagnosis, respectively (Table 2). Proxies that included inhaled corticosteroids (nr 2, 4, and 6) yielded therefore higher PPVs (0.76, 0.84, and 0.87, respectively) for the identification of children with an asthma diagnosis. In addition, including ≥2 prescriptions for anti-asthma drugs increased the PPV as well (Fig. 1).

### Validation of the medication proxies for the identification of children diagnosed with atopic dermatitis

In Table 5, the sensitivity and PPV of the six medication proxies for the identification of children diagnosed with atopic dermatitis are shown. When a capture period of 1 year was applied, medication proxy 9, receiving at least 1 prescription for dermal corticosteroids, yielded the highest sensitivity of 0.80 (95 % CI 0.77–0.82). However, only 39 % of the identified children had a registered diagnosis of atopic dermatitis (PPV 0.39, 95 % CI 0.37–0.41). Table 3 shows that of the children who got prescribed dermal corticosteroids, 37.6 % had been diagnosed with the ICPC code for contact dermatitis/other dermatitis. Medication proxy 13, receiving at least 4 prescriptions for dermal steroids within a year, yielded a higher PPV of 0.75 (95 % CI 0.65–0.85). However, only 12 % of the children diagnosed with atopic dermatitis were detected.

### Validation of the medication proxies for the identification of children diagnosed with allergic rhinitis

In Table 5, the sensitivity and PPV of the nine medication proxies for the identification of children diagnosed with allergic rhinitis are shown. When a capture period of 1 year was applied, medication proxy 15, receiving at least 1 prescription for a anithistamine, yielded the highest sensitivity of 0.72 (95 % CI 0.68–0.76). However, only 23 % of the identified children had a diagnosis of allergic rhinitis (PPV 0.23, 95 % CI 0.20–0.25). Medication proxy 16, receiving at least 1 prescription for nasal antihistamine, yielded a higher PPV of 0.87 (95 % CI 0.81–0.93). However, only 22 % of the children diagnosed with allergic rhinitis were detected. Table 4 showed that of the children who got prescribed nasal antihistamines, 27.2 % received the diagnosis asthma without a concomitant diagnosis of allergic rhinitis.

**Table 5:** Accuracy measures of medication proxies for the identification of children (0–10 years) diagnosed with asthma, atopic dermatitis, and allergic rhinitis

		Half-year capture period		1-year capture period		Within study period	
						(max 10 years)	
Medication proxies		Sensitivity (95 % CI)	PPV (95 % CI)	Sensitivity (95 % CI)	PPV (95 % CI)	Sensitivity (95 % CI)	PPV (95 % CI)
Asthma							
1	≥1 inhaled anti-asthma drugs	0.92 (0.90–0.94)	0.54 (0.51–0.57)	0.92 (0.90–0.94)	0.54 (0.51–0.57)	0.92 (0.90–0.94)	0.54 (0.51–0.57)
2	≥1 inhaled steroid	0.62 (0.59–0.66)	0.76 (0.73–0.80)	0.62 (0.59–0.66)	0.76 (0.73–0.80)	0.62 (0.59–0.66)	0.76 (0.73–0.80)
3	≥2 inhaled anti-asthma drugs	0.65 (0.62–0.69)	0.75 (0.72–0.79)	0.69 (0.66–0.73)	0.74 (0.70–0.77)	0.75 (0.72–0.78)	0.71 (0.68–0.74)
4	≥1 inhaled steroid + ≥1 inhaled other anti-asthma drug	0.52 (0.49–0.55)	0.85 (0.82–0.88)	0.54 (0.50–0.57)	0.84 (0.81–0.87)	0.55 (0.51–0.58)	0.83 (0.80–0.87)
5	≥3 inhaled anti-asthma drugs	0.45 (0.42–0.49)	0.83 (0.80–0.87)	0.54 (0.50–0.57)	0.82 (0.79–0.85)	0.63 (0.60–0.67)	0.80 (0.77–0.83)
6	≥2 inhaled steroids	0.43 (0.39–0.46)	0.87 (0.84–0.91)	0.46 (0.43–0.49)	0.87 (0.83–0.90)	0.49 (0.46–0.53)	0.85 (0.82–0.89)
7	≥4 inhaled anti-asthma drugs	0.33 (0.30–0.36)	0.88 (0.84–0.92)	0.44 (0.40–0.47)	0.86 (0.83–0.90)	0.53 (0.50–0.57)	0.83 (0.80–0.87)
8	≥5 inhaled anti-asthma drugs	0.23 (0.20–0.26)	0.90 (0.86–0.94)	0.35 (0.32–0.38)	0.90 (0.87–0.93)	0.48 (0.45–0.51)	0.87 (0.83–0.90)
	Atopic dermatitis						
9	≥1 dermal steroid	0.80 (0.77–0.82)	0.40 (0.38–0.42)	0.80 (0.77–0.82)	0.40 (0.38–0.42)	0.80 (0.77–0.82)	0.40 (0.38–0.42)
10	≥1 immunosuppressant	0.01 (0.01–0.02)	0.71 (0.49–0.92)	0.01 (0.01–0.02)	0.71 (0.49–0.92)	0.01 (0.01–0.02)	0.71 (0.49–0.92)
11	≥2 dermal steroids	0.33 (0.30–0.36)	0.60 (0.56–0.64)	0.40 (0.37–0.43)	0.60 (0.56–0.64)	0.50 (0.47–0.53)	0.57 (0.54–0.60)
12	≥3 dermal steroids	0.14 (0.12–0.16)	0.71 (0.64–0.77)	0.19 (0.16–0.21)	0.67 (0.61–0.72)	0.31 (0.28–0.34)	0.64 (0.60–0.69)
13	≥4 dermal steroids	0.06 (0.04–0.07)	0.74 (0.64–0.84)	0.12 (0.10–0.14)	0.75 (0.65–0.85)	0.20 (0.18–0.23)	0.71 (0.65–0.76)
14	≥5 dermal steroids	0.03 (0.02–0.04)	0.74 (0.61–0.88)	0.06 (0.04–0.07)	0.78 (0.71–0.85)	0.15 (0.13–0.17)	0.77 (0.71–0.83)
	Rhinitis						
15	≥1 systemic antihistamine	0.72 (0.68–0.76)	0.23 (0.20–0.25)	0.72 (0.68–0.76)	0.23 (0.20–0.25)	0.72 (0.68–0.76)	0.23 (0.20–0.25)
16	≥1 nasal antihistamine	0.22 (0.18–0.26)	0.87 (0.81–0.93)	0.22 (0.18–0.26)	0.87 (0.81–0.93)	0.22 (0.18–0.26)	0.87 (0.81–0.93)
17	≥1 nasal steroid	0.36 (0.32–0.41)	0.43 (0.38–0.48)	0.36 (0.32–0.41)	0.43 (0.38–0.48)	0.36 (0.32–0.41)	0.43 (0.38–0.48)
18	≥2 systemic antihistamines, nasal antihistamines or nasal steroids	0.53 (0.49–0.58)	0.47 (0.42–0.51)	0.59 (0.54–0.64)	0.43 (0.39–0.47)	0.67 (0.62–0.71)	0.40 (0.36–0.43)
19	≥3 systemic antihistamines, nasal antihistamines or nasal steroids	0.34 (0.29–0.38)	0.61 (0.54–0.67)	0.40 (0.35–0.45)	0.57 (0.51–0.62)	0.52 (0.47–0.57)	0.50 (0.46–0.55)
20	≥2 nasal steroids	0.16 (0.12–0.19)	0.59 (0.50–0.68)	0.18 (0.14–0.22)	0.60 (0.51–0.68)	0.20 (0.16–0.24)	0.57 (0.49–0.65)
21	≥4 systemic antihistamines, nasal antihistamines or nasal steroids	0.19 (0.15–0.23)	0.67 (0.58–0.75)	0.27 (0.23–0.31)	0.63 (0.56–0.70)	0.40 (0.36–0.45)	0.58 (0.52–0.64)
22	≥5 systemic antihistamines, nasal antihistamines or nasal steroids	0.13 (0.10–0.16)	0.71 (0.60–0.81)	0.18 (0.15–0.22)	0.65 (0.56–0.73)	0.34 (0.30–0.39)	0.62 (0.56–0.69)

Variation of the capture period for the detection of prescriptions had more effect on sensitivity values (maximum change 400 % for proxy 14) than that on the PPVs (maximum change of 18 % for proxy 20) for the identification of children diagnosed with asthma, atopic dermatitis, and allergic rhinitis.

Additional analyses, in which the population was stratified by age, showed that accuracy measures of the proxies designed for the identification of children diagnosed with atopic dermatitis were higher in the age group 0–5 years (Table 6). On the contrary, accuracy measures of the proxies designed for the identification of children diagnosed with asthma and allergic rhinitis were higher in the age group 6–10 years (Table 6).

**Table 6:** Accuracy measures of medication proxies for the identification of children diagnosed with asthma, atopic dermatitis, and allergic rhinitis for children aged 0–5 and children aged 5–10

		1-year capture period			
		0–5 years		6–10 years	
	Medication proxies	Sensitivity (95 % CI)	PPV (95 % CI)	Sensitivity (95 % CI)	PPV (95 % CI)
	Asthma				
1	≥1 inhaled anti-asthma drugs	0.90 (0.87–0.92)	0.48 (0.45–0.51)	0.94 (0.91–0.96)	0.71 (0.67–0.75)
2	≥1 inhaled steroid	0.58 (0.54–0.62)	0.71 (0.67–0.75)	0.68 (0.63–0.72)	0.84 (0.80–0.88)
3	≥2 inhaled anti-asthma drugs	0.64 (0.60–0.68)	0.67 (0.63–0.71)	0.75 (0.71–0.79)	0.85 (0.81–0.89)
4	≥1 inhaled steroid + ≥1 inhaled other anti-asthma drug	0.49 (0.45–0.53)	0.79 (0.75–0.84)	0.57 (0.52–0.61)	0.89 (0.85–0.93)
5	≥3 inhaled anti-asthma drugs	0.49 (0.45–0.53)	0.77(0.73–0.81)	0.58 (0.53–0.63)	0.88 (0.84–0.92)
6	≥2 inhaled steroids	0.43 (0.39–0.47)	0.81 (0.77–0.86)	0.51 (0.46–0.56)	0.92 (0.89–0.96)
7	≥4 inhaled anti-asthma drugs	0.39 (0.35–0.43)	0.82 (0.77–0.87)	0.50 (0.45–0.54)	0.91 (0.87–0.95)
8	≥5 inhaled anti-asthma drugs	0.30 (0.26–0.34)	0.85 (0.81–0.90)	0.40 (0.35–0.45)	0.94 (0.90–0.97)
	Atopic dermatitis				
9	≥1 dermal steroid	0.78 (0.75–0.81)	0.45 (0.43–0.48)	0.77 (0.72–0.82)	0.29 (0.26–0.33)
10	≥1 immunosuppressant	0.01 (0.00–0.02)	0.78 (0.51–1.05)	0.02 (0.00–0.03)	0.63 (0.29–0.96)
11	≥2 dermal steroids	0.40 (0.36–0.43)	0.65 (0.61–0.70)	0.37 (0.31–0.42)	0.48 (0.41–0.54)
12	≥3 dermal steroids	0.19 (0.16–0.22)	0.72 (0.66–0.78)	0.15 (0.11–0.19)	0.52 (0.41–0.62)
13	≥4 dermal steroids	0.12 (0.10–0.14)	0.71 (0.60–0.82)	0.09 (0.06–0.12)	0.71 (0.57–0.85)
14	≥5 dermal steroids	0.06 (0.04–0.07)	0.71 (0.60–0.82)	0.04 (0.02–0.06)	0.72 (0.52–0.93)
	Rhinitis				
15	≥1 systemic antihistamine	0.71 (0.63–0.78)	0.12 (0.09–0.14)	0.70 (0.65–0.75)	0.33 (0.30–0.37)
16	≥1 nasal antihistamine	0.11 (0.05–0.16)	0.78 (0.59–0.97)	0.25 (0.21–0.30)	0.90 (0.83–0.96)
17	≥1 nasal steroid	0.20 (0.13–0.26)	0.28 (0.19–0.37)	0.38 (0.33–0.44)	0.45 (0.39–0.51)
18	≥2 systemic antihistamines, nasal antihistamines or nasal steroids	0.47 (0.38–0.55)	0.23 (0.18–0.28)	0.61 (0.56–0.67)	0.55 (0.50–0.60)
19	≥3 systemic antihistamines, nasal antihistamines or nasal steroids	0.30 (0.22–0.38)	0.35 (0.26–0.44)	0.42 (0.37–0.47)	0.64 (0.58–0.71)
20	≥2 nasal steroids	0.11 (0.05–0.16)	0.61 (0.41–0.81)	0.19 (0.15–0.23)	0.57 (0.47–0.66)
21	≥4 systemic antihistamines, nasal antihistamines or nasal steroids	0.16 (0.10–0.22)	0.36 (0.24–0.49)	0.33 (0.25–0.35)	0.68 (0.61–0.76)
22	≥5 systemic antihistamines, nasal antihistamines or nasal steroids	0.11 (0.06–0.17)	0.42 (0.26–0.58)	0.21 (0.17–0.25)	0.70 (0.61–0.79)

### Sensitivity analyses

**Fig. 2:**
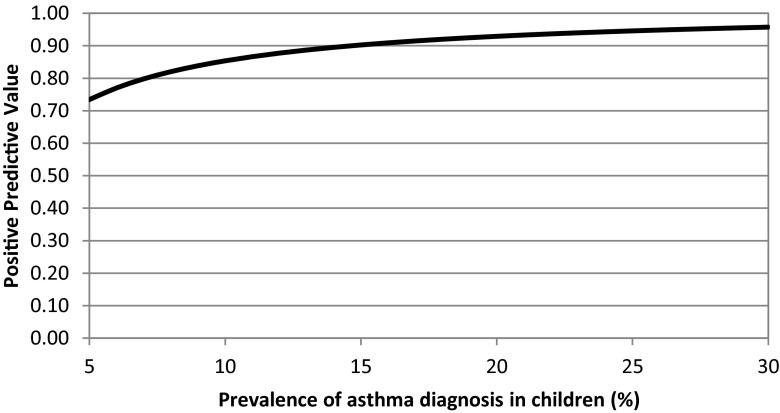
Sensitivity of the PPV for childhood asthma as a function of the prevalence for a range of values [20]. Positive predictive values were derived from the proxy ≥2 inhaled steroids in a year

The results of the sensitivity analyses on the PPVs of the proxies ≥2 inhaled steroids in a year were presented in Fig. 2. This figure showed that the PPV for the identification of children diagnosed with asthma was accurate for a reasonable range of asthma prevalence numbers [20].

## Discussion

The current study showed that accuracy measures of the medication proxies for asthma were relatively high, especially in children older than 5 years. This suggests that children in this age group diagnosed with asthma can be identified reliably with prescription data. On the contrary, sensitivity and positive predictive values of the proxies designed for the identification of children diagnosed with atopic dermatitis and rhinitis were lower. The use of prescription data for the identification of children diagnosed with atopic dermatitis and allergic rhinitis is therefore questionable. This study provides different medication proxies to aid various epidemiological studies with the identification of children diagnosed with atopic diseases.

### Interpretation and comparison with literature

Of the different allergic diseases, medication proxies designed for the identification of asthmatic children yielded highest accuracy measures, especially for children who were diagnosed beyond the age of 5. The evaluation of asthma in young children is complicated by the lack of objective lung function measurements [9], explaining the slightly better prediction of an asthma diagnosis in the older age group. In addition, anti-asthmatic medication was prescribed for other respiratory conditions, like acute bronchitis and cough. Results showed that a better distinction between different respiratory conditions can be made with the inclusion of inhaled corticosteroids in the proxy. In addition, medication proxies can yield higher PPVs if the proxy includes multiple prescriptions for inhaled asthma drugs. Accuracy measures of our medication proxies for the identification of children diagnosed with asthma were comparable with other validation studies [3, 7, 8]. However, our study showed better accuracy measures in the age group 0–5 than any of the previously performed studies when the proxy with ≥2 prescriptions for inhaled steroids was applied. Though asthma cannot be diagnosed objectively before the age of 5, in the Netherlands, it is a common practice to diagnose children with recurrent wheeze as being asthmatic. This may explain the slightly better accuracy measures in the current study. In addition, the majority of the medication proxies presented in the current study included the total group of anti-asthma medication rather than specific subtypes of anti-asthma medication. According to previous studies, the PPV may even be higher if medication proxies included more specific subgroups of anti-asthma drugs [3]. The medication proxies for the detection of children with atopic dermatitis yielded lower accuracy measures. This can mainly be explained by the use of dermal steroids for other indications than atopic dermatitis, like the indication contact dermatitis/other dermatitis (ICPC S88). Since atopic dermatitis is often treated for a longer duration than other types of dermatitis, differentiation between indications can only be made with a medication proxy that resembles a long-term treatment (≥4 prescriptions for dermal steroids). The medication proxy of 4 prescriptions for dermal corticosteroids within the total time of the study period yielded an acceptable PPV of 0.75 in the age group 0–10 and an even higher PPV in the age group 0–5. However, the sensitivity of this proxy was really low (0.12). Hence, consideration should be made if its use is feasible, since only a really small percentage of children with a diagnosis of atopic dermatitis will be detected. This medication proxy may only be applied in large databases. Only one previous study validated a medication proxy for the identification of children diagnosed with atopic dermatitis and reported a PPV of 0.45 [8]. The PPV of the current study is much higher (0.75). This can mainly be explained by the fact that the medication proxy designed in the current study resembled long-term treatment.

This is the first study that validated medication proxies designed for the identification of children diagnosed with allergic rhinitis. The medication proxy ≥1 prescription for nasal antihistamines yielded a high PPV of 0.87 (95 % CI 0.81–0.93). However, similar to the medication proxy for atopic dermatitis, the sensitivity is low (0.22) and its use is questionable.

### Strengths and limitations

The major strength of the current study is the validity testing of a wide range of medication proxies for the identification of children diagnosed with different atopic diseases. Previous studies that validated medication proxies for the identification of children with asthma mainly validated proxies applicable in etiological research and focused on PPVs. Though many studies focus on etiological research, proxies which prioritize sensitivity over PPV may also be useful. To our knowledge, this is the first study that validated medication proxies for the identification of children diagnosed with allergic rhinitis and the second to validate the identification of children diagnosed with atopic dermatitis with prescribing data.

The RNG database is representative for the Netherlands as a whole, and results are generalizable to the Dutch childhood population. Since prescribing guidelines differ between countries, it should be noted that findings from our study may be at least in part specific for the situation in the Netherlands. Though misclassification can occur in every database, the coding of ICPC and ATC codes by the GPs in the RNG database was proven to be accurate by previous studies [7, 13]. Though GPs in the RNG database have been trained specifically to work with the coding system, behavior of diagnosing and prescribing may vary between practices and influence accuracy measures of the medication proxies. Nonetheless, additional analyses detected no difference between the practices (percentages of truly positive identified asthma patients varied from 8.6 until 11.1 % between practices) in identifying diagnosed asthmatic children with the medication proxy ≥1 prescription for anti-asthma drugs (data on file).

### Implications for future research

The selection of a particular medication proxy will always depend on the focus of interest of a study and the available data sources. Though studies that require maximizing one accuracy measure completely at the expense of another are rare, there are situations in which one accuracy measure may be more important than the other [10]. In the following scenarios, we discuss the applicability of the presented proxies for future research.

The sensitivity of a proxy may be given more importance if the goal is to identify all patients with a certain condition in a population. The proxy ≥1 prescription for anti-asthmatic drugs detects 92 % of the children with an asthma diagnosis. As a consequence, 46 % of the included cases are false positives. This may not be a problem if additional verification, like a personal interview, takes place after inclusion. Another scenario that requires a proxy with a high sensitivity is if the aim is to assess the full range of disease outcomes rather than only the most severe. Less sensitive proxies may detect only the severe outcomes, so results may not be generalizable to the whole patient population. This may be important when, for example, a study focusses on the effectiveness of asthma treatment. A proxy with a low sensitivity may fail to detect the effectiveness of asthma treatment that works for mild but not for severe asthma cases. For atopic dermatitis and allergic rhinitis, medication proxies that yielded acceptable sensitivity values yielded low PPVs. The applicability of these proxies for future research is questionable, since more than half of the included patients will be false positives. Therefore, additional verification of the condition should take place after inclusion.

The PPV of a proxy may be given more importance over the sensitivity of a proxy when a study aims to identify only patients that truly have the condition, rather than be representative of all persons that have the condition. In etiological studies, researchers want to ensure that the children that are defined as cases do actually have the disease [21]. A proxy with a low PPV includes more false positives and may fail to detect an association between the exposure and the outcome, since this would bias the effect estimate towards the null. For asthma, the proxy ≥2 inhaled steroids within a year can be applied since it yielded a high PPV (0.87) and still half of the patients diagnosed with asthma were detected. However, the proxies that yielded satisfactory PPVs for atopic dermatitis and allergic rhinitis yielded low sensitivity values. Hence, it should be considered if its use is feasible, since only a really small percentage of children with a diagnosis of these conditions will be detected. Since the PPV is an indirect accuracy measure and dependent on the sensitivity and specificity, it cannot be influenced directly by the researcher. In addition, the PPV is influenced by the prevalence of the condition, which may vary between different populations. However, sensitivity analyses of varying prevalence of the disease on the PPV of the proxy showed that PPVs were satisfactory over a reasonable range of prevalence numbers of asthma (Fig. 2).

Since the specificity and NPVs were artificially high due to the large number of non-allergic patients included in the study population, we did not calculate these accuracy measures and no recommendations can be made for these accuracy measures.

In conclusion, this study showed that children diagnosed with asthma can be identified reliably with a range of medication proxies. The use of prescription data for the identification of children diagnosed with atopic dermatitis and allergic rhinitis is questionable, since sufficient PPVs were only yielded in combination with low sensitivity values. Data collection for childhood patients is challenging, and prescription databases may provide convenient and easily available sources. The broad spectrum of medication proxies presented in this study may aid various epidemiological studies with the identification of children diagnosed with allergic disorders in the future.
